# Etiological approach of chylothorax in Babol, northern Iran

**Published:** 2017

**Authors:** Novin Nikbakhsh, Mohammad Zamani, Askari Noorbaran, Ali Naghshineh, Danial Rastergar-Nejad

**Affiliations:** 1 Cancer Research Center, Babol University of Medical Sciences, Babol, Iran.; 2 Student Research Committee, Babol University of Medical Sciences, Babol, Iran; 3 Babol University of Medical Sciences, Babol, Iran.

**Keywords:** Chylothorax, Lymph, Thoracic duct, Trauma, Surgery

## Abstract

**Background:**

Chylothorax results from leakage of lymph in the pleural cavity because of thoracic duct injury which is associated with severe metabolic disorders. The aim of this study was to evaluate the rate of chylothorax and its causes among hospitalized patients in Shahid Beheshti Hospital of Babol city, North of Iran.

**Methods:**

In this cross-sectional study, all patients with chylothorax admitted to the surgery department of Shahid Beheshti Hospital during 2002-2015 were included. Information including gender, age, duration of symptoms, laboratory findings, causes of disease and the type of treatment were extracted from the patients' records.

**Results:**

Of the 42 patients, 27 (64.3%) were men and 15 (35.7%) were women. The mean age of the study population was 51.03±16.95. The most common clinical symptoms were dyspnea (66.7%) and dyspnea with cough (21.4%), respectively. In all patients, the pleural fluid triglyceride level was greater than 110 mg/dl, whereas the presence of lymphatic in pleural fluid was eventful in 18 (42.8%) patients. The causes of the disease were traumatic (54.8%), non-traumatic (38.1%) and unknown (7.1%), which were not significantly correlated with gender. Nineteen (45.2%) patients were operated, 16 (38.1%) patients received supportive therapy, and 7 (16.7%) patients had the treatment of the underlying conditions and then supportive therapy.

**Conclusion:**

According to the results, trauma was the most common cause of chylothorax. Therefore, identification and control of the traumatic factors seem to be the steps to prevent and reduce the chylothorax incidence and its complications.

Lymph fluid contains large amounts of chylomicron, triglyceride, cholesterol, fat-soluble vitamins, lymphocytes, immunoglobulins, enzymes and products of digestion. The rate of approximately 2.4 liters of chyle is passed daily by lymphatic system. Chylothorax is the accumulation of lymphatic fluid in the pleural space due to damage or rupture of the thoracic duct (the largest lymph vessel which is passed through the thoracic cavity and discharged into the subclavian vein) (1). Its prevalence varies from 0.5% to 2% in thoracic surgery and also even to 3% in esophagectomy with mediastinal lymphadenectomy (2, 3). Chyloma is a chyle collection that is rarely detected, but some of its clinical manifestations such as swelling, which occurred in the supraclavicular fossa, may be associated with severe chest pain, dyspnea, and tachycardia. In some cases, chyloma can be found incidentally in x-ray (4, 5). In chronic cases in which fluid effusion is not noticed and controlled, chylothorax can be associated with malnutrition, weight loss and muscle wasting (6).

Chylothorax is categorized as traumatic and non-traumatic causes. Diseases including sarcoidosis, hemangiomatosis, retrosternal goiter, benign tumor, heart failure, yellow nail syndrome, malignancy, and idiopathic factors are related to non-traumatic causes (4). With regard to traumatic chylothorax, iatrogenic causes such as thoracic surgery, head and neck surgery, radiation, and also, non-iatrogenic factors like stab, shot, childbirth, coughing, vomiting with pressure, and blunt thoracic trauma or vertebral are discussed (1, 7-9). To diagnose chylothorax, computed tomography scan (CT scan) of the abdomen and chest, lymphangiography, definitive thoracentesis tests and laboratory analysis of the pleural fluid (pleural fluid triglyceride level, presence of chylomicron in pleura, and the presence of lymphatic in pleura fluid) are used by the physicians (1). 

Chylothorax treatment can be divided into 3 groups: treatment of underlying conditions (10), conservative therapy (11), and surgery (12-14). Considering the clinical importance of this complication and its high mortality rates (75%) due to malnutrition, dehydration, and immune imbalance (15), identifying the causes leading to chylothorax will be a step to prevent and reduce its effects. So far, there is not any report regarding the incidence rate of chylothorax in Babol. Therefore, the aim of this study was to investigate the rate, causes and symptoms of chylothorax in patients who were admitted to Shahid Beheshti Hospital in Babol.

## Methods

In this cross-sectional retrospective study, all patients with chylothorax hospitalized in the surgery department of Shahid Beheshti Hospital of Babol, one of the active trauma centers in the province, during 2002-2015 were included. The necessary data including sex, age, clinical symptoms such as cough, dyspnea, dyspnea with cough, duration of clinical symptoms, laboratory data (e.g. the triglyceride level of pleural fluid, the presence of lymph in the pleural fluid), and the cause of chylothorax and treatment protocol were extracted from the patients' records. 

The diagnosis of chylothorax was based on: 1) the triglyceride level of pleural fluid which was over 110 mg/dl, 2) the confirmation of the presence of lymph in pleural fluid during lymphangiography and/or surgery (typical appearance of chylothorax).

The study was approved by the Ethics Committee of Babol University of Medical Sciences and Health Services (No: 2006). For statistical analysis, the software SPSS V16 was used. The collected data were compared between genders using the chi-square test. A p-value less than 0.05 was considered as statistically significant.

## Results

Of the 42 studied patients, 27 (64.29%) were men and 15 (35.71%) were women. The mean age of the study population was 51.03±16.95 that ranged from 20 to 79 years. The average age of men and women were 48.86±17.11 and 55.8±16.41 years, respectively. The most common clinical symptoms were dyspnea in 28 (66.7%) patients and dyspnea with cough in 9 (21.4%) patients (table 1). The duration of the symptoms was 18.4 on average**,** which was in the range of 1 to 90 days. This quantity for men and women was 9. 9 to 14.2 and 25.9 to 29.2 days, respectively. With respect to the laboratory findings, the triglyceride level of pleural effusion was greater than 110 mg/dl in all patients, whereas in the lymphangiography, the lymph was observed in only18 (42.8%) of the patients' pleural fluid. The cause of chylothorax in 23 (54.8%) patients was traumatic, in 16 (38.1%) patients was non-traumatic, and in 3 (7.1%) patients was unknown (table 1).

**Table 1:** Distribution of symptoms, diagnostic criteria and etiology of chylothorax in patients based on gender.

**Variables**	**Male (%)** **N=27**	**Female (%)** **N=15**	**pvalue**
**The prevalence of symptoms**			
Dyspnea	18 (66.7)	10 (66.7)	> 0.99
Dyspnea with cough	5 (18.5)	4 (26.6)	
Asymptomatic	4 (14.8)	1 (6.7)	
**Diagnostic criteria**			
Pleural fluid triglyceride level >110mg/dl	27 (100)	15 (100)	0.14
Presence of lymph in the pleural fluid	15 (55.5)	3 (20)	
**Etiology**			
Traumatic	18 (66.7)	5 (33.3)	0.99
Non-traumatic	8 (29.7)	8 (53.3)	
Unknown	1 (3.6)	2 (13.4)	

The frequency of traumatic and non-traumatic causes of chylothorax is separately shown in table 2. In relation to the traumatic etiology, blunt trauma was mostly seen in 60.9% patients, and regarding the non-traumatic causes, lung cancer was the most common cause observed in 31.25% of the patients. There was not any significant relationship between gender and symptoms in the completed evaluation. In addition, the association between sex and type of test was not significant (P=0.14). Furthermore, no significant correlation was found between gender and the causes of chylothorax (P=0.99).

**Table 2:** Distribution of traumatic and non-traumatic causes of chylothorax

**Etiology**	**Frequency (%)**
**Traumatic**	
Blunt trauma	14 (60.9)
Surgical trauma	5 (21.7)
Penetrating trauma	4 (17.4)
**Non-traumatic**	
Lung cancer	5 (31.2)
Lymphoma	4 (25)
Leukemia	3 (18.8)
Stomach cancer	2 (12.5)
Tuberculosis	2 (12.5)

Nineteen (45.2%) patients needed surgery for the treatment. On the other hand, 16 (38.1%) patients received only the conservative therapy for the treatment. For 7 (16.7%) patients, first the treatment of the underlying condition, and then, the conservative treatment were performed. During the process of treatment, 32 (76.2%) patients recovered completely, 6 (14.3%) patients had partial response, and 4 (9.5%) patients did not recover which caused the death of 3 (7.1%) of them with cancer. All six patients who had partial recovery, likewise one of the 4 patients who did not recover and was alive during the study, underwent non-surgical treatment due to the underlying disease (disease course and anesthesia intolerance). Additionally, of the 3 patients who died, 2 patients with the underlying factor of lung cancer had a non-surgical procedure due to lack of tolerance. While another patient with the underlying disease of esophagus cancer who had a gastric pull-up 3 years after the last surgery in which surgery intervention was selected for the treatment, unfortunately the patient died.

## Discussion

Chylothorax is a rare condition and a special kind of effusion cirrhosis which has different reasons. Sharkey and Rao in their review study stated that the most common causes of chylothorax were thoracic surgery, non-surgical trauma, cancers (frequently lymphoma), and some infections such as tuberculosis (1). In the study by Zabeck et al., it was found that surgery is responsible for chylothorax in 45% of cases, and in other 55% patients, non-surgical factors (including malignancy, disorders of lymph nodes, liver cirrhosis, and other causes) have been described as chylothorax causes (16). In a retrospective study by Maldonado et al., it was indicated that chylothorax occurred in 51% of cases after surgery (17). The study by Doerr et al. revealed that trauma is the most common cause (49.8%),and different medical conditions and other unknown factors had a lower frequency (5). 

In one study, Marts et al. evaluated 29 patients and declared that chylothorax was seen more on the right side and less frequently on the left side. The incidence of this complication was more frequent after the surgery of congenital heart disease, and other factors such as esophageal surgery, trauma and thoracic surgery were less involved (18). In our study, trauma was the most common cause of chylothorax (54.8%). Because of the importance of Shahid Beheshti Hospital of Babol as one of the active site of trauma in the province, we witnessed an increase in trauma patients in this center.

The present study consisted of 42 patients (27 (64.3%) men and 15 (35.7%) women). The mean age of the study population was 51.03±16.95. In the research of Maldonado et al., it was reported that the studied people consisted of 37 (50%) men and 37 (50%) women, and the age of the patients ranged from 20 to 93 years (17). In other study, 29 patients consisting of 16 (55%) males and 13 (45%) females were evaluated in which the mean age of the patients was 20.1 ranged 5 days to 76 years (18). Considering that the etiology of chylothorax can vary in different populations, it is considered that the prevalence of chylothorax can change among different genders and age groups.

In the current study, the most common clinical symptoms were dyspnea and dyspnea with cough that nearly corresponds to the results of other studies. A survey on 6 children (consisted of 4 boys and 2 girls) with an idiopathic chylothorax, revealed that the patients' manifestations were cough (4 patients), tachypnea (4 patients), asthenia (5 patients), abdominal pain (2 patients), and bronchitis (1 patient) (19). Moreover, McGrath et al. in their review, proposed that inflammation (swelling) in the supraclavicular fossa, severe chest pain, dyspnea, and tachycardia are as the most common symptoms (4). Furthermore, Xu et al.'s study showed that dyspnea, edema, abdominal pain, and weight lost were the symptoms of chylothorax incidence (20). In our study, the mean duration of clinical presentations of chylothorax was 18.4 days, ranged 1-90 days. Shah et al., in Pennsylvania, also stated that the mean time of chylothorax diagnosis was 5 days, and the mean duration of hospitalization was 17 days (21). 

On the other hand, another report showed that the duration of symptoms before diagnosis was 7.5 weeks that ranged from 1 day- 4.5 years (5). About the diagnostic criteria, Soto-Martinez and Massie in their review revealed that thoracentesis of pleural fluid was helpful, but they announced that its results can be changed by the type of diet. They have also made the definitive diagnosis on the results of laboratory tests of pleural fluid and the biochemical tests associated with Sudan staining (10). On the other hand, Maldonado et al. expressed that the value of diagnostic laboratory tests of pleural fluid triglyceride was less than 110 mg/dl (17). 

In the present study, the pleural fluid triglyceride level was more than 110 mg/dl in all patients for the diagnosis. For management and treatment of chylothorax, various ways have been recommended. Sharkey and Rao proposed that the ligation of the thoracic duct at the diaphragm level is the original treatment, especially for those that chyle drainage will be taken more than 3 weeks. Nonetheless, in their study, they alluded the use of somatostatin drug as useful as a long-acting treatment and a supportive treatment after nutrition therapy (1). 

Soto-Martinez and Massie have brought up that drainage, changes in diet, and then treatment with somatostatin as the primary treatment in the pediatric chylothorax (10). In the review by Nairet et al., the surgical treatment was considered as the most appropriate treatment options when the incidence factor was trauma or surgery (6). In our study, according to the underlying factor and the patient's status, the treatment was divided into three types: 1) treatment of underlying condition, 2) supportive therapy, and 3) surgical treatment, in which surgical treatment has been a great success. Considering that multiple factors are involved in the occurrence of chylothorax, the use of treatment type will be different in variant studies. In this regard, more surveys should be conducted to select the best type of treatment. There are some limitations regarding our article. For example, this study was conducted only in one hospital and it is needed that further investigations also be done in other health centers in Babol, with more subjects and variables.

In conclusion, due to the major disadvantages of chylothorax, the heavy costs of the treatment in the hospitals and patients plus that given trauma was the most common cause of chylothorax in our study, it seems necessary that a series of policies be implemented to prevent trauma and subsequently to reduce chylothorax incidence rate and its complications and costs.
